# Public Response to Scientific Misconduct: Assessing Changes in Public Sentiment Toward the Stimulus-Triggered Acquisition of Pluripotency (STAP) Cell Case via Twitter

**DOI:** 10.2196/publichealth.5980

**Published:** 2017-04-20

**Authors:** Alberto Gayle, Motomu Shimaoka

**Affiliations:** ^1^ Mie University Graduate School of Medicine Tsu Japan

**Keywords:** scientific misconduct, retraction of publication as a topic, mass media, social media, public opinion, public policy, data mining, publication, stem cells, Japan

## Abstract

**Background:**

In this age of social media, any news—good or bad—has the potential to spread in unpredictable ways. Changes in public sentiment have the potential to either drive or limit investment in publicly funded activities, such as scientific research. As a result, understanding the ways in which reported cases of scientific misconduct shape public sentiment is becoming increasingly essential—for researchers and institutions, as well as for policy makers and funders. In this study, we thus set out to assess and define the patterns according to which public sentiment may change in response to reported cases of scientific misconduct. This study focuses on the public response to the events involved in a recent case of major scientific misconduct that occurred in 2014 in Japan—stimulus-triggered acquisition of pluripotency (STAP) cell case.

**Objectives:**

The aims of this study were to determine (1) the patterns according to which public sentiment changes in response to scientific misconduct; (2) whether such measures vary significantly, coincident with major timeline events; and (3) whether the changes observed mirror the response patterns reported in the literature with respect to other classes of events, such as entertainment news and disaster reports.

**Methods:**

The recent STAP cell scandal is used as a test case. Changes in the volume and polarity of discussion were assessed using a sampling of case-related Twitter data, published between January 28, 2014 and March 15, 2015. Rapidminer was used for text processing and the popular bag-of-words algorithm, SentiWordNet, was used in Rapidminer to calculate sentiment for each sample Tweet. Relative volume and sentiment was then assessed overall, month-to-month, and with respect to individual entities.

**Results:**

Despite the ostensibly negative subject, average sentiment over the observed period tended to be neutral (−0.04); however, a notable downward trend (*y*=−0.01 *x* +0.09; *R* ²=.45) was observed month-to-month. Notably polarized tweets accounted for less than one-third of sampled discussion: 17.49% (1656/9467) negative and 12.59% positive (1192/9467). Significant polarization was found in only 4 out of the 15 months covered, with significant variation month-to-month (*P*<.001). Significant increases in polarization tended to coincide with increased discussion volume surrounding major events (*P*<.001).

**Conclusions:**

These results suggest that public opinion toward scientific research may be subject to the same sensationalist dynamics driving public opinion in other, consumer-oriented topics. The patterns in public response observed here, with respect to the STAP cell case, were found to be consistent with those observed in the literature with respect to other classes of news-worthy events on Twitter. Discussion was found to become strongly polarized only during times of increased public attention, and such increases tended to be driven primarily by negative reporting and reactionary commentary.

## Introduction

### Background

With the rise of social network services (SNS), all news events, no matter how large or small, have become subject to intense public scrutiny and debate [1,2]. Of course, this has gone on in some form or another since the advent of civilization. However, the democratization, reach, and consequence of public scrutiny has never before been realized to the degree seen today [3-5]. Indeed, although assessment of public opinion has traditionally been the domain of pollsters and social scientists, social media analytics are increasingly being seen as a reliable alternative [6]. With a large and increasingly diverse demographic base, Twitter has been shown to be reasonably representative in terms of demographics [7,8] and public sentiment [9], especially with respect to breaking news [10-12].

Recent investigations into communication on Twitter have uncovered common, generalizable patterns in the way sentiment changes in response to the emergence of notable events—namely, that increases in public attention are coincident with increases in negative sentiment [13]. Such patterns follow known dynamics associated with media sensationalism [14,15] and have been observed across a broad spectrum of mass media topics, including entertainment, sports, business, politics, and natural disasters [16]. Sensationalism has also been found to be a problem in the reporting of medical science [17]. This is of particular concern given the profound and lasting impact on the direction of public policy that sensationalist reporting might have [18]. And although studies have examined the role of the traditional news media in shaping public opinion as it relates to medical science and policy [19], no studies to date have explored whether such dynamics would apply to the presumably expert-driven communications on Twitter.

One area of particular interest is scientific misconduct, particularly in the areas of academic and medical science. Scientific misconduct concerns more than just a given researcher or institution; damage to public perception of, and goodwill toward scientific research itself is a driving concern [20]. Most academic research institutions derive the bulk of their research budgets from public spending, and so a loss of reputation can have a direct and far-ranging impact. Academic institutions invest heavily in anticipation of future academic trends and research demands [21]; consequently, unanticipated changes in public policy or funding may result in large, unrecoupable capital expenditures and lost opportunity [22]. Understanding the specific dynamics governing public response to reports of scientific misconduct on social media is therefore invaluable.

### Stimulus-Triggered Acquisition of Pluripotency Cell Case

Here, we assess and define the patterns according to which public sentiment may change in response to reports of academic scientific misconduct on Twitter. This study focuses on public response to a recent and widely covered case of scientific misconduct—the stimulus-triggered acquisition of pluripotency (STAP) cell case that occurred in Japan in 2014 [23]. The STAP cell case is used in this study, as this represents the major scientific misconduct in the era of SNS and was well mentioned by the mainstream media such as Nature, Science, New York Times, Cable News Network (CNN), and British Broadcasting Corporation (BBC). In this case, media reports focused on Ms Obokata, an upcoming biochemist, who attracted as much attention for her achievements, as for her gender and youth. Attention also focused on notable coauthors, Dr Y Sasai, Dr T Wakayama, and Dr C Vacanti, as well as the sponsoring institution, Riken.

Here, we have demonstrated that Twitter response to the STAP case tended to generally stay neutral, but specifically skew negative as discussion polarity and volume increased. Our results are consistent with those observed in studies covering other topics of interest [24]. These findings suggest that changes in public sentiment toward any major event—cases of scientific misconduct included—might be as much a function of the attention received as it is a function of the theme or merits of any specific case.

## Methods

### Data Collection

For the purpose of this analysis, an event timeline was constructed based on primary reports and press-releases [25,26]. Where such data were lacking, secondary sources were vetted and compiled [23,27-29]. All data obtained were used and reported according to the terms and privacy policies of the respective data sources. Where such no such policy existed, data were assumed to be public domain.

Twitter data (“Tweets”) were obtained directly from Apple’s now defunct subsidiary, Topsy [30]. According to Twitter, “a Tweet...is a message of 140 characters or less that is public by default” [31]. For the purposes of this study, the data obtained was handled according to established ethical precedent regarding public domain social media content, that is, consent of the authors was neither required nor obtained [32]. Our search covered a full 14 months, beginning with the day of Riken’s press release concerning STAP (January 29, 2014) and ending March 31, 2015. Prior research has demonstrated that datasets derived using a limited set of focused keywords are suitable for the purpose of analyzing public sentiment regarding a specific issue or brand [33]. In order to minimize collection of irrelevant Tweets, the following minimum set of search criteria were chosen: obokata OR 小保方 OR #obokata OR #小保方. Other, potentially relevant terms, such as “STAP” and “Riken” were excluded at this stage after having been found to return results that were likely to be either redundant or irrelevant.

An automated platform for downloading and processing Tweets was developed using RStudio [34]. Using this platform, we obtained a sample set consisting of all English-language Tweets covering the relevant timeframe polled at a rate of n=100 Tweets every 3 h. All content as well as associated metadata (eg, author name, date, and number of retweets) were downloaded. This initial, raw dataset contained N=12,925 Tweets in total. Full-text was retained for qualitative analysis.

### Data Processing

Initial processing of data was conducted programmatically within RStudio. This included formatting and transformation of the downloaded data into tables. Downloaded tables were saved and processed using Excel 2013. This included the programmatic identification and removal of all Tweets containing non-English characters or text. In addition, all Tweets were manually checked to remove irrelevant or spam posts, resulting in a final dataset of n=9467 Tweets total.

For the purpose of sentiment classification, RapidMiner version 6.3, Enterprise Edition (Rapid-I GmbH, Dortmund, Germany) was used. The following preprocessing steps were followed (Table 1). These steps are common across the many domains in which text-mining is applied [35], with minimal variation.

**Table 1:** Text processing steps.

Steps	Description
Tokenize	Parse every tweet into separate, single-element tokens (ie, words or word-parts)
Transform cases	Makes all text lower case to facilitate data processing
Filter tokens by length	Removes tokens consisting of less than 2 characters
Filter English stop words	Removes common, low-information particles (eg, “the”) and punctuation marks
Filter tokens by content	Removed hashtags and other message-irrelevant tokens such as “http”
Stemming (WordNet)	Algorithm for identifying and groups tokens as lemmas, to facilitate processing
Generate n-grams	Generates list of all two-, three-, or four-word token combinations (ie, phrases)
Word vector creation	Generate metric indicative of the measuring the important of each word in a tweet
Pruning	Remove tokens that appear in less than 1% or more than 80% of documents

This processing generated weighted word vectors, representing the weighted distribution of each processed token or n-gram within a given Tweet. Word vector statistics were calculated using the term frequency-inverse document frequency (TF-IDF) weighting scheme. TF-IDF emphasizes the importance of key but not uncommon terms [36,37] and has been demonstrated to improve the performance of text-mining tasks [38]. TF-IDF is calculated as follows: TF-IDF=tf*log (N/df), where *tf* is the frequency of a term within a given document, *df* is the frequency across all documents, and *N* is the number of documents total [39].

To evaluate sentiment for each Tweet, the SentiWordNet 3.0 extension was used within Rapidminer. SentiWordNet is a well-established sentiment analysis protocol and has been cited by almost 1000 (988) journal publications as of the date of this writing, according to Google Scholar search. SentiWordNet assigns three sentiment scores (“positive,” “negative,” and “objective”) to each word, based on a generalized classification system developed by the authors using a combination of manual and automated sentiment scoring algorithms [40]. SentiWordNet’s “bag-of-words” methodology has been demonstrated to be reliable for document-level sentiment analysis, with aggregate-level performance roughly on par with more sophisticated methods, including human coding [41].

For this analysis, nouns were omitted from sentiment calculation. Recent studies have demonstrated that, for automated sentiment analyses, nouns are not likely to provide additional, reliable information [42]. And in topics with terminology that is either uncommon or uncommonly applied, this is even more the case—especially when using a general purpose lexicon such as SentiWordNet [43]. All terms were, however, retained for topic-level analysis. The sentiment of each Tweet was then calculated by aggregating the scores of all relevant word tokens, as determined using SentiWordNet.

Scores were thus assigned for each Tweet, ranging from −1 to +1, based on the estimated degree of negative or positive sentiment. These scores are reported in unstandardized form. For the purpose of statistical analysis and visualization, scores were then standardized, to produce a distribution with mean of zero (*x̄*=0) and standard deviation of one (*σ*_x̅_=1). All Tweets with standardized scores less than −1 were labeled “negative,” whereas those with standardized scores greater than +1 were labeled “positive;” Tweets with standardized scores less than +1 but greater than −1 were labeled “neutral.”

A support vector machine (SVM) analysis was then used to identify the terms and phrases that were most commonly associated with each respective sentiment label. SVM is a computational method that derives a classification scheme based on the degree to which the various input cases (ie, word vectors) predict a given binary class (eg, positive or negative sentiment or “mentions Sasai” or null) [44]. All input terms (and term combinations, ie, “n-grams”) can thus be assessed in terms of “importance” with respect to a given label [45]. Conceptually, this is similar to a logistic regression; however, the computation is far more computationally intensive [46,47]. In addition, Tweets mentioning Ms Obokata, Dr Sasai, and the Riken institution were labeled accordingly; associated terms or phrases were also extracted via SVM.

### Data Analysis and Visualization

Once the Twitter data were processed as described, the data were exported to Microsoft Excel for further processing using the Pivot Table function. Sentiment as well as sampled Tweet volume were aggregated and indices were calculated for all relevant sub- and cross-tables. These tables were then used to generate visualizations either directly in Microsoft Excel or using ggplot2 and ggtern in RStudio. In cases where a given table or visualization suggested a time-trend or association with respect to aggregate sentiment or Tweet volume, statistical significance was assessed using chi-squared and Tukey’s post hoc (1-way analysis of variance, ANOVA) tests. A GLM model (with Bonferroni correction) was used to test month-to-month mean difference versus previous months; 2-way ANOVA was used to compare metrics for individual entities. SPSS Statistics version 23 (IBM Corp) was used for all statistical tests.

## Results

### Analysis of Sentiment

**Figure 1:**
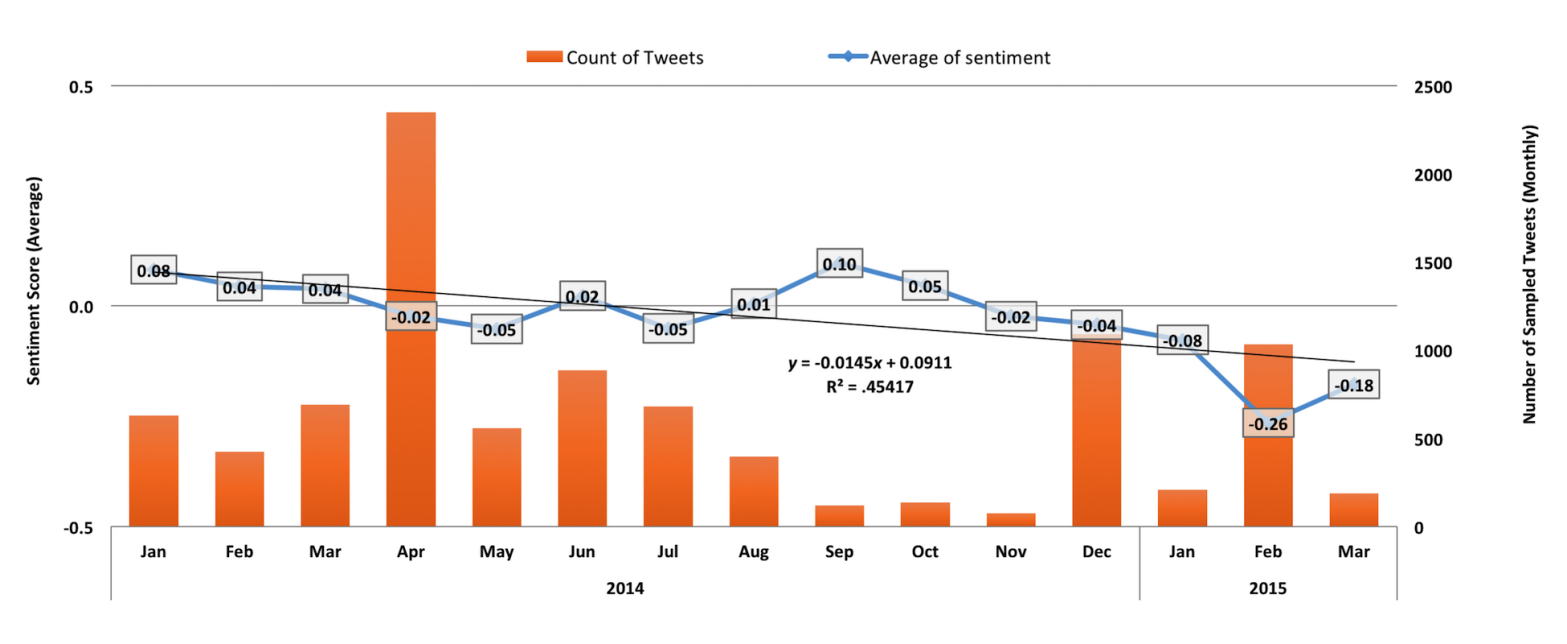
Volume and average sentiment over time. Sentiment score calculated using unweighted aggregate sentiment scores found in the SentiWordNet database, for each valid token in each Tweet. For this analysis, verb, adjectives, and adverbs were considered valid for the purpose of sentiment scoring. Volume is based on number of Tweets retrieved per sampling interval. Sentiment increasingly negative over time; one key exception corresponds with the tragedy surrounding Dr Sasai (August to October 2014). Volume is driven by major events.

**Figure 2:**
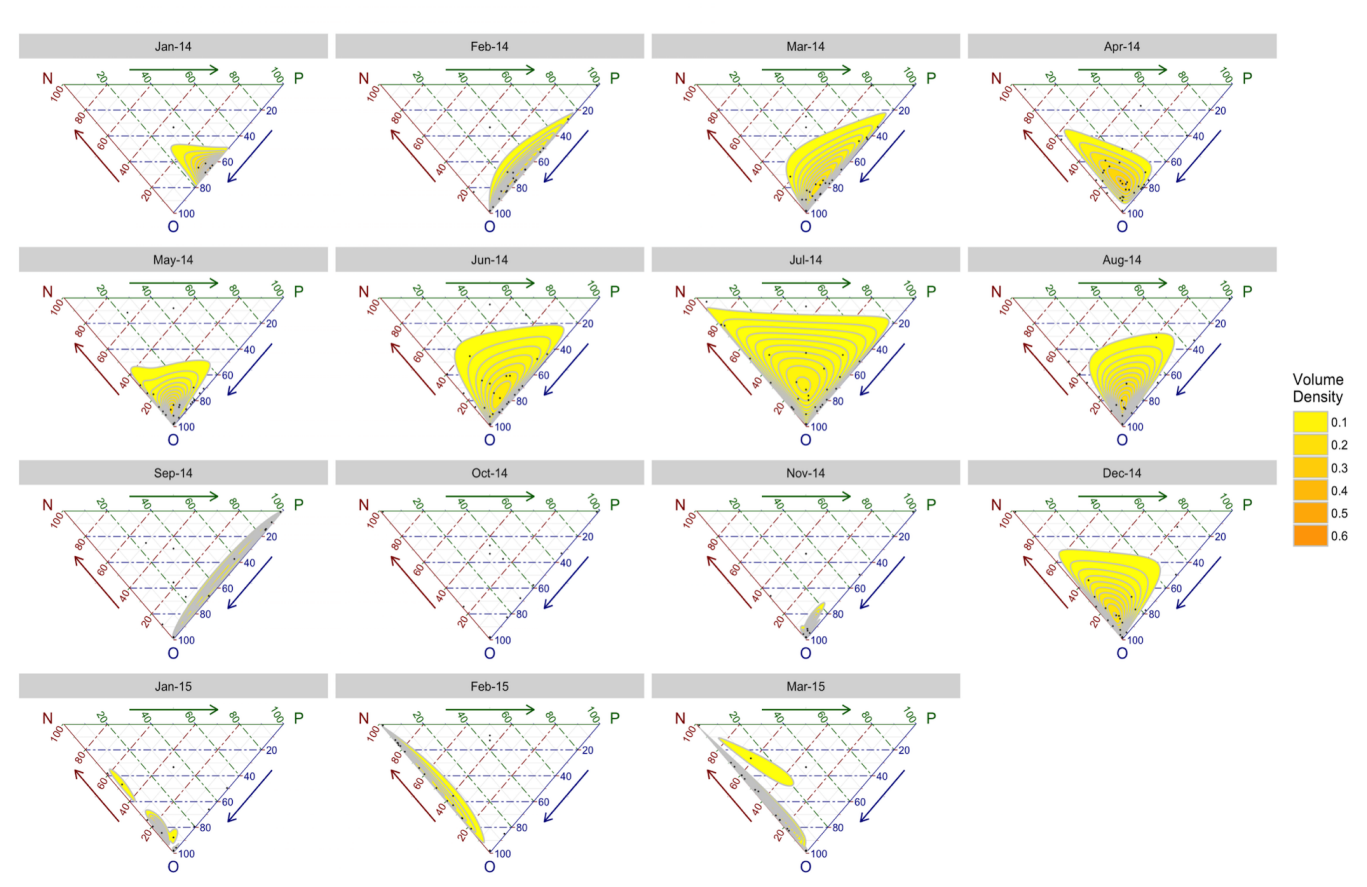
Month-to-month trinary sentiment or volume density chart. Density plot calculated based on the proportion of negative (N: top left), positive (P: top right), and objective or nonpolarized (O: bottom center) discussion volume (represented by the unlabeled data points). Volume density is calculated via isometric log ratio transformation.

Over the 15-month period covered, overall sentiment was found to be −0.037 on average, with a notable downward trend (Figure 1). Over this same period, sampled Tweets averaged 631.1 Tweets per month, with a maximum of 2349 Tweets (April 2014; 372.2% index) and a minimum of 75 Tweets (November 2014; 11.9% index). One-way ANOVA with Tukey’s honest significant difference (HSD) confirmed significant month-to-month variation with respect to sentiment (Table 2). Overall, discussion tended to be mostly neutral (69.92%), with positive and negative discussion being far less prevalent (12.59% and 17.49%, respectively) overall. Discussion month-to-month tended to be mostly objective; however, when polarized, discussion tended negative (Figure 2). Chi-squared tests confirmed observed differences to be significant in this respect as well (Table 3).

**Table 2:** Tukey’s post hoc test for significance (1-way analysis of variance, ANOVA). Italicized values indicate significance *P*<.05.

Year	Month	2014												2015			
		Jan	Feb	Mar	Apr	May	Jun	Jul	Aug	Sep	Oct	Nov	Dec	Jan	Feb	Mar
2014	Jan	0.000	−*0.039*	−*0.044*	−*0.105*	−*0.135*	−*0.060*	−*0.134*	−*0.078*	0.015	−0.036	−*0.106*	−*0.125*	−*0.160*	−*0.348*	−*0.260*
	Feb	*0.039*	0.000	−0.005	*0.067*	−*0.096*	−0.021	−*0.095*	−0.039	0.053	0.002	−0.067	*0.087*	−*0.121*	−*0.309*	−*0.222*
	Mar	*0.044*	0.005	0.000	−*0.062*	−*0.091*	−0.016	−*0.090*	−0.034	*0.059*	0.008	−0.062	−*0.081*	−*0.116*	−*0.304*	−*0.217*
	Apr	*0.105*	*0.067*	*0.062*	0.000	−*0.030*	*0.045*	−*0.029*	0.028	*0.120*	*0.069*	−0.001	−0.020	−*0.054*	−*0.242*	−*0.155*
	May	*0.135*	*0.096*	*0.091*	*0.030*	0.000	*0.075*	0.001	*0.057*	*0.150*	*0.099*	0.029	0.010	−0.025	−*0.213*	−*0.125*
	Jun	*0.060*	0.021	0.016	−*0.045*	−*0.075*	0.000	−*0.074*	−0.018	*0.075*	0.024	−0.046	−*0.065*	−*0.100*	−*0.288*	−*0.200*
	Jul	*0.134*	*0.095*	*0.090*	*0.029*	−0.001	*0.074*	0.000	*0.056*	*0.149*	*0.098*	0.028	0.009	−0.026	−*0.214*	−*0.126*
	Aug	*0.078*	0.039	0.034	−0.028	−*0.057*	0.018	−*0.056*	0.000	*0.093*	0.041	−0.028	−*0.047*	−*0.082*	−*0.270*	−*0.183*
	Sep	−0.015	−0.053	−*0.059*	−*0.120*	−*0.150*	−*0.075*	−*0.149*	−*0.093*	0.000	−0.051	−*0.121*	−*0.140*	−*0.175*	−*0.363*	−*0.275*
	Oct	0.036	−0.002	−0.008	−*0.069*	−*0.099*	−0.024	−*0.098*	−0.041	0.051	0.000	−0.070	−*0.089*	−*0.123*	−*0.312*	−*0.224*
	Nov	*0.106*	0.067	0.062	0.001	−0.029	0.046	−0.028	0.028	*0.121*	0.070	0.000	−0.019	−0.054	−*0.242*	−*0.154*
	Dec	*0.125*	*0.087*	*0.081*	0.020	−0.010	*0.065*	−0.009	*0.047*	*0.140*	*0.089*	0.019	0.000	−0.035	−*0.223*	−*0.135*
2015	Jan	*0.160*	*0.121*	*0.116*	*0.054*	0.025	*0.100*	0.026	*0.082*	*0.175*	*0.123*	0.054	0.035	0.000	−*0.188*	−*0.101*
	Feb	*0.348*	*0.309*	*0.304*	*0.242*	*0.213*	*0.288*	*0.214*	*0.270*	*0.363*	*0.312*	*0.242*	*0.223*	*0.188*	0.000	*0.087*
	Mar	*0.260*	*0.222*	*0.217*	*0.155*	*0.125*	*0.200*	*0.126*	*0.183*	*0.275*	*0.224*	*0.154*	*0.135*	*0.101*	−*0.087*	0.000

**Table 3:** Tukey’s post hoc test for homogenous subsets (1-way analysis of variance, ANOVA).

Year	Month	N	Subset for alpha=.05								
			1	2	3	4	5	6	7	8	9
2015	February	1034	−.2648								
	March	187		−.1774							
	January	209			−.0768						
2014	May	558			−.0521	−.0521					
	July	680			−.0510	−.0510					
	December	1092			−.0422	−.0422	−.0422				
	November	75				−.0230	−.0230	−.0230			
	April	2349				−.0224	−.0224	−.0224			
	August	395					.0052	.0052	.0052		
	June	887						.0230	.0230		
	March	691							.0391	.0391	
	February	424							.0443	.0443	
	October	136							.0467	.0467	
	January	630								.0829	.0829
	September	120									.0978
Significance			*P*>.99	*P*>.99	*P*=.52	*P*=.77	*P*=.07	*P*=.09	*P*=.21	*P*=.14	*P*>.99

Both sets of trends were assessed against the timeline of events to determine whether sentiment and volume varied according to actual, real-world events. Findings are reported in terms of average sentiment, volume index (ratio of monthly or average volume), and positivity index (ratio of positive to negative volume). Multimedia Appendix 1 presents a summary timeline. A brief analysis is as follows.

### Timeline Analysis and Analysis of Key Events

#### January 2014: STAP Announced to Much Fanfare; Obokata Hailed

On January 29, 2014, a letter [48] and research article [49] describing a “unique and unexpected cellular reprogramming phenomenon, called stimulus-triggered acquisition of pluripotency (STAP)” were published in Nature (ISSN: 0028-0836) by a team from the Riken Center for Developmental Biology in Japan and collaborators. These papers claimed that “stressors reprogram mammalian somatic cells, resulting in the generation of pluripotent cells”—a claim which challenged the very tenets of cell biology. Beginning one day before publication, the lead author, Ms Haruko Obokata, was hailed as a rising star in a series of media events and press releases [50].

For the 3 days comprising the month of January 2014 (January 29-31), average sentiment was found to be second highest among all months covered (0.083; *P*<.001, versus grand mean). In terms of volume, January indexed at par (99.8% index; 630/631.1); however, positive index was exceptionally high (3583.3%; 215/6). Discussion hails the “breakthrough” discovery; initial skepticism is present but muted.

#### February 2014: Doubts Begin to Surface; Riken Launches Investigation

Average sentiment decreased significantly, but remained overall positive (0.044; *P*=.004). In terms of volume, February indexed below par (67.2% index; 424/631.1); positivity also remained high (888.9%; 80/9). Discussion continues to focus on the merits and implications of STAP; however, strong doubts begin to take hold.

- Initial concerns about possible figure manipulation first voiced on PubPeer (February 4) [51].
- STAP coauthor, Dr Charles Vacanti, posts images claimed to be human STAP cells (February 5) [52].
- Riken subsequently launches an investigation into “alleged irregularities” for both papers [53].

#### March 2014: Calls for Retraction Mount; Obokata Increasingly Implicated

Average sentiment decreased slightly, but not significantly (0.039; *P*>.99). However, volume began to increase (109.5% index; 691/631.1); discussion remained positive, albeit to a lesser degree (387.1%; 120/31). Allegations of possible misconduct increasingly take center stage; some antiskepticism still remains.

- “Essential technical tips for STAP...” published by Ms Obokata, Dr Sasai, and Dr Niwa on March 3 [54].
- STAP coauthor, Dr Wakayama, breaks from others and proposes retraction of both papers [55].
- In an interim report, the Riken investigation team finds inappropriate handling of data [56].

#### April 2014: Obokata Declared Guilty Amid Shake-Up at Riken; Public Interest Spikes

Discussion volume reached its peak (372.2% index; 2349/631.1); however, discussion took on a more negative tone. Average sentiment decreased significantly (−0.022; *P*<.001), with a positivity now at 88.7% index (276/311). Riken’s finding of misconduct, problems with the investigation team, and Ms Obokata’s claims of innocence dominate; discussion centers on the drama between actors and institution, not the science.

- Riken finds Ms Obokata guilty of “two instances of research misconduct” in the STAP work [57].
- Ms Obokata holds a press conference in order to rebut Riken’s conclusions [58].
- Nature publishes a strongly worded editorial on science policy in Japan, citing STAP case [59].

#### May 2014: Retraction Appears Inevitable; Obokata, However, Still Defiant

Average sentiment continues the negative trend (−0.052; *P*=.003). A substantial drop in volume was also observed (88.4% index; 558/631.1), accompanied by a sharp drop in positivity as well (57%; 29/51). Fueled by reports of prior, scathing rejections by Science and Cell, discussion now focuses on the nature of the experiments and results; Riken’s initial rejection of Ms Obokata’s appeal also a hot topic.

- Ms Obokata, under pressure to retract both papers, agrees to retract only the letter (May 28).
- Other senior authors continue to negotiate with Ms Obokata regarding the remaining article [60].

#### June 2014: STAP Papers Retracted; End Now in Sight, Calls for Punitive Action Emerge

Driven by a sharp increase in positivity (245%; 137/56) and overall discussion (140.5% index; 887/631.1), average sentiment became slightly positive (0.023; *P*<.001). Despite calls for punitive action against the senior investigators, STAP retraction is discussed in neutral terms. Decision to suspend disciplinary process against Ms Obokata and allow her to join STAP reproduction efforts is discussed favorably.

- STAP coauthor, Dr Wakayama, presents genetic evidence refuting the existence of STAP cells [61].
- Ms Obokata and coauthors finally agree to retract both papers published in Nature [62].
- Riken reform committee recommends restructuring of Center for Developmental Biology [63].

#### July 2014: STAP Discussion Takes on a Lighter—and Somewhat Derisive—Tone

Average sentiment was once again negative (−0.051; *P*<.001), with a positivity index of 67.8% (97/143) against volume only slightly above par (107.7% index; 680/631.1). Discussion focuses on the fallout of the STAP retraction.

- On July 3, the two Nature papers reporting the STAP cells are retracted [64,65].
- Ms Obokata sustains injuries while being pursued by television reporters [66].
- Concurrent with Riken’s investigation of the STAP case, Waseda University begins investigation of alleged plagiarism in Ms Obokata’s doctoral dissertation [67]; Ms Obokata retains PhD, for now.

#### August 2014: Sasai’s Suicide Draws Muted Response

Discussion volume was subpar (62.6% index; 395/631.1); despite a positivity index of 115% (45/39), average sentiment was nevertheless mixed (0.005; *P*<.001). Discussion focuses on the apparent suicide of Dr Sasai; commentary is reserved, though some take the opportunity to critique the academic culture in Japan.

- On August 5, Dr Sasai, a STAP coauthor, found dead at the Riken center due to apparent suicide.
- Dr Sasai leaves behind a note addressed to Ms Obokata, urging her to verify existence of STAP [68].
- A STAP coauthor, Dr Niwa, announces his lab’s failure to replicate STAP results (August 27) [69].

#### September 2014: Discussion More Sympathetic, Pensive as STAP Retrial Continues

Driven by a large increase in positivity (1575%; 63/4), average sentiment improved considerably (0.098; *P*<.001). Nevertheless, discussion volume was remarkably low (19% index; 120/631.1). No single topic stands out; discussion instead touches on themes ranging from Ms Obokata’s mentoring to the role of the media.

- Vacanti et al release new STAP protocol; addition of adenosine triphosphate (ATP) now asserted to be key (September 3) [70].
- Dr Endo publishes a report suggesting that STAP cells may have been embryonic stem cells (September 21) [71].

#### October 2014: Waseda University’s Threat to Revoke PhD Draws Mixed Response

In October 2014, average sentiment decreased significantly, but remained overall positive (0.047; *P*=.23. Despite low overall discussion volume (21.5% index; 136/631.1), positivity was comparably high (1267%; 38/3). Discussion volume, however, remained overall low. Discussion mostly focuses on Waseda University’s demand that Ms Obokata correct her dissertation [72]; some see this as an “opportunity.”

#### November 2014: Attention Focused Elsewhere as STAP Retrial Draws to a Close

In November 2014, discussion volume fell to an all-time low (11.9% index; 75/631.1). Despite above average positivity (150%; 3/2), average sentiment continued to trend negative (−0.023; *P*=.06). Discussion centers on speculation regarding the STAP replication efforts. No other coherent themes emerge.

#### December 2014: Riken Unexpectedly Halts STAP Retrial; Obokata Resigns

In December 2014, average sentiment decreased slightly, but not to a significant degree (−0.042; *P*>.99). However, a large increase in discussion volume (173% index; 1092/631.1) precipitated a sharp drop in positivity (54.2%; 71/131). Ms Obokata’s failure to replicate STAP, along with her abrupt resignation attracts the most discussion; “disgraced” is the now most commonly used term to describe Ms Obokata.

- Riken halts STAP verification experiments, announcing them to have failed [73].
- Ms Obokata resigns from her position at Riken [74].

#### January 2015: Obokata Accused of Stealing Materials; Criminal Charges Threatened

In January 2015, the downward trend in average sentiment continued—albeit not to a significant degree (−0.08; *P*=.10). Discussion volume (33.1% index; 209/631.1) and positivity were both subpar (23%; 6/26). Discussion focuses on allegations of criminal wrongdoing.

#### February 2015: Riken Openly Discusses Punitive Measures; Guardian Piece Attracts Mixed Response

In February 2015, a large uptick in discussion (163.8% index; 1034/631.1) drove a precipitous decline in average sentiment (−0.26; *P*<.001) and positivity (1.0%; 8/766). Discussion focuses on the following:

- Riken’s announcement of penalties related to the STAP research and publication.
- Riken’s public announcement of plans to pursue criminal charges against Ms Obokata [75].
- The Guardian piece, “What pushes scientists to lie,” [76] is highly cited during this period.

#### March 2015: Riken Announces Intent Not to Sue Obokata

In March 2015, average sentiment improves slightly but remains extremely negative (−0.18; *P*<.001). Positivity remains low (5%; 4/78) amid low overall discussion volume (29.6% index; 187/631.1). Despite a few supportive messages, discussion overwhelmingly highlights Ms Obokata’s continuing woes.

- Riken’s decision not to sue Ms Obokata [77] garners mixed attention.
- However, reported demands that she return publication-related expenses [78] draws derision.

### Sentiment Surrounding Various, Individual Stakeholders

Significant differences were found with respect to the sentiment surrounding various parties. Tweet data were aggregated according to whether Ms “Obokata,” Dr “Sasai,” or (inclusive) the “Riken” institute were mentioned. Overall, sentiment surrounding Ms Obokata and the Riken institute was found to be consistent with broader trends (−0.04 and −0.03, respectively; no significant difference). However, sentiment toward Dr Sasai was found to be significantly more positive overall (0.03; *P*<.04), with visible trends coinciding with relevant events; but these results were not statistically significant (Figure 2).

**Figure 3:**
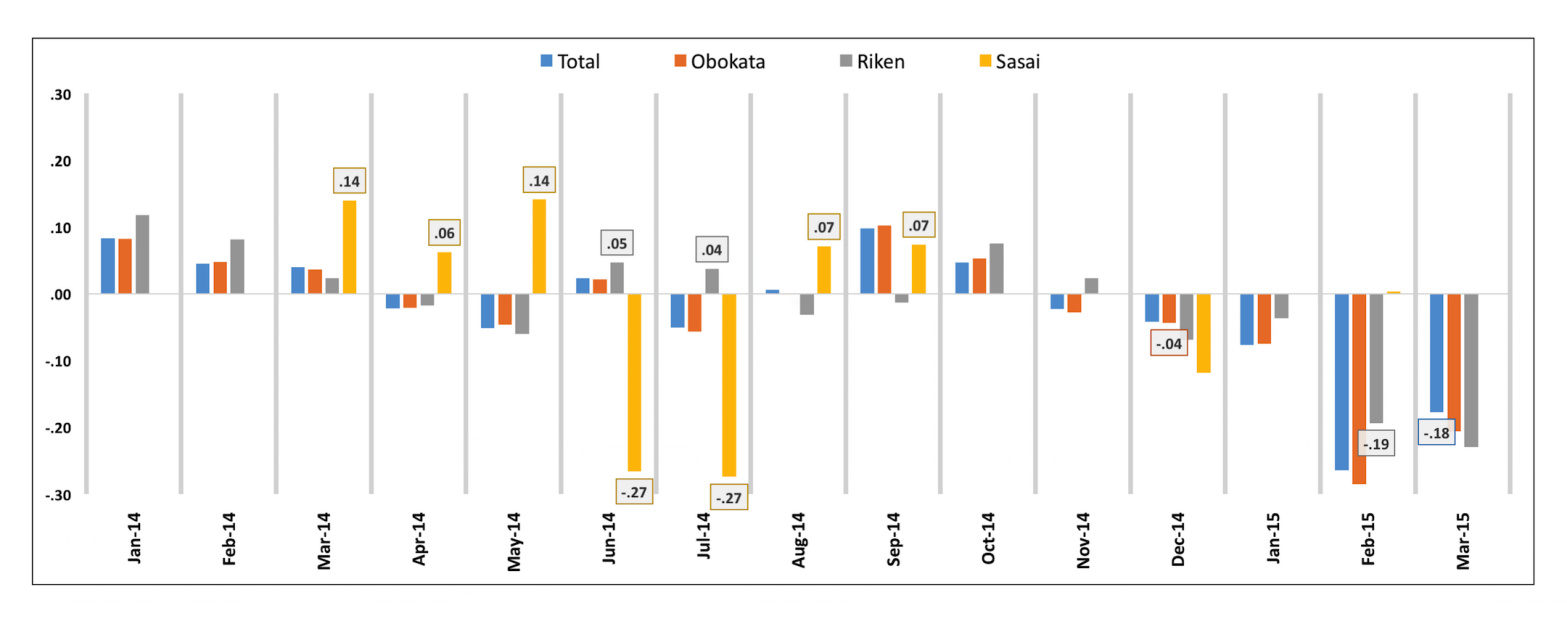
Sentiment comparison for key actors. Month-to-month sentiment for key figures and entities corresponds with associated timeline events. Month-to-month sentiment scores were independently aggregated for Tweets mentioning Ms Obokata, Dr Sasai, or Riken. Data labels shown where mean differences are significant versus total.

Dr Sasai initially received minimum attention. However, once allegations of misconduct began to emerge, Dr Sasai’s continued, public support of Ms Obokata becomes increasingly associated with discussion that was significantly more favorable (0.14, 0.06, and 0.14 respectively, in March 2014, April 2014, and May 2014; *P*<.001, *P*=.05, *P*=.02 respectively). Indeed, discussion mentioning Dr Sasai remained positive even as sentiment toward Ms Obokata (−0.02 and −0.05 respectively, in April 2014 and May 2014) and Riken (−0.02 and −0.06 respectively, in April 2014 and May 2014) became decidedly negative. Incident to and just before retraction of the STAP papers, discussion increasingly turned to the role of Ms Obokata’s mentors and institutional culpability. Accordingly, sentiment associated with mentions of Dr Sasai fell precipitously during this period (−0.27 in both June 2014 and July 2014; *P*=.09 and *P*<.001, respectively). By contrast, sentiment associated with Riken was positive during the same timeframe (0.05 and 0.04 respectively, in June 2014 and July 2014; *P*=.01 and *P*=.62, respectively; Figure 3).

These trends, however, reversed in August and September of that year. Coincident with and following the tragedy surrounding Dr Sasai, the sentiment associated with mentions of Dr Sasai became positive (0.07 and 0.07 respectively, in August 2014 and September 2014; *P*=.04 for both), whereas sentiment associated with Riken followed the opposite trend (−0.03 and −0.01 respectively, in August 2014 and September 2014; *P*<.001 and *P*=.03, respectively).

### Content Analysis

A simplified “grounded theory” approach [79] was used to examine the generalizable themes that were most commonly expressed within this dataset. For this purpose, entities specific to this case were omitted. Each Tweet was manually categorized according to major topic or polarity-driving theme. Coding was based on an analysis of the terms and concepts within a given Tweet that correspond with the degree and direction of polarity assigned by the SentiWordNet algorithm. Categories were added, merged, and/or eliminated progressively, as Tweets were reviewed. Higher-level codes were manually assigned to similarity clusters in an iterative fashion, to uncover overarching themes. Tweets were allowed to be assigned to multiple coding classes. The primary themes thus found to be driving polarity were the following: (1) “unfocused negativity—outrage or mockery,” (2) “cynicism toward academic science,” (3) “defense of academic science,” and (4) “miscellaneous conspiracy theories.”

## Discussion

### Principal Findings

Only a few studies have covered the publicity of science using SNS [80,81]; and thus, very little is currently known about how public sentiment may change in response to reported cases of scientific misconduct. A study conducted on behalf of the UK House of Lords found that, although public interest in science is high, “a culture of...secrecy...invites suspicion” [82]. However, whether such suspicions are based on rational or justifiable criticism is still a matter of controversy [83]. In addition, little is currently known as to whether and how news related to scientific misconduct is relevant to or impacts public opinion on a broad scale.

This study found that STAP-related discussion volume varied significantly month-to-month, coincident with new events. Furthermore, we found that month-to-month sentiment was generally neutral or of mixed composition, tending to skew negative when polarized. This is consistent with previous findings concerning the characteristics of public sentiment as expressed on Twitter [84]. In this study, it was noted that such increases in polarity generally corresponded with large increases in overall volume, that is, major events attracted more attention, most of which tended negative. It is interesting to note that such increases in volume and corresponding negativity were generally short-lived; positivity, on the other hand, tended to be much less prone to such fluctuation.

In addition, this analysis found that sentiment surrounding various stakeholders differed significantly with respect to specific events. Of particular note is the sentiment surrounding Dr Sasai, the researcher whose tragic fate was found to correspond with an increase in positive sentiment. The relationship between the death of a key stakeholder in a public crisis and subsequent improvement of the public mood—from criticism to sympathy—has been covered in the Japanese literature in the 1980s [85] and also in a recent Time article reporting Dr Sasai’s death [86]. And in this study, an analysis of public sentiment, expressed via social media, was able to detect and observe this effect.

The results presented here provide an important case study for understanding the impact of scientific misconduct on public sentiment. The coverage received by the STAP cell case can be attributed to many factors, but the instrumentality of social media cannot be ignored. Although this manuscript was undergoing review, a related study was published that provided rudimentary analysis of the print and social media coverage of the STAP cell case in Japan [87]. This study confirmed that print coverage tended to lag behind social media coverage—a finding which supports other reports suggesting that news initiated and driven by public interest may indeed influence public opinion [9,88], or at least mainstream reporting. Here, we have presented a more robust perspective by including all major timeline events in the STAP cell case and by employing a well-established, objective method for sentiment analysis. This research is the first to establish the patterns according to which public opinion evolves in response to reports of scientific misconduct in the popular press.

The question still remains, however, whether sentiment expressed on Twitter regarding future cases of misconduct will accurately reflect overall public sentiment. Prior research has demonstrated that the Twitter medium most effectively influences or reflects public response with respect to high-volume events or crises [89]. And some research has even suggested superiority to traditional polling [6] in some respects. For this study, the case covered was considered to be one of the largest and most impactful cases of scientific misconduct in recent memory [27]. Moreover, this case is the first and only major scientific misconduct case to occur in an era where social media coverage and documentation is so ubiquitous. Indeed, much of the initial concerns and evidence regarding STAP originated in social media [90-93]. And recent research and commentary on evolving mass media trends suggests that social media is increasingly becoming both an initiator and driver of public attention and news cycles [88]. Consequently, we expect the impact of future cases of major scientific misconduct to be generalizable, using social media metrics in the fashion demonstrated here [94,95].

### Limitations

The text- and sentiment-analysis procedures employed in this study were robust and well-validated. The reported metrics are limited by the analytical processes that were used to derive them from the text. In this case, SentiWordNet was used to obtain sentiment scores. Reported sentiment scores are thus limited by the accuracy and precision of the SentiWordNet database with respective material covered. In addition, volume estimations were based on and limited by the distribution characteristics of the sample obtained from the data provider. Furthermore, the reported metrics are estimations, as is the case with all sampling-based analytical approaches. That having been said, the analytical and data retrieval methods used are well established and have been verified to be sufficiently robust for such analyses [92,94]. Future studies would benefit from larger sample sizes and more precise sentiment estimation methods; however, based on previous studies, improvements are expected to be marginal.

### Conclusions

This study represents the first objective analysis of public response to a major case of scientific misconduct. This study observes and tracks changes in public sentiment over a 15-month sequence of events associated with the STAP cell case, which was one of the most publicized cases of major scientific misconduct in recent memory. Here, we demonstrated that public response to this particular case tended to be generally neutral or of mixed composition, particularly during times of lower public attention. This was observed in the large majority of months covered in this study. Also observed was that sentiment tended to skew negative as discussion polarity and volume increased. These findings are generally consistent with those observed in the literature with respect to major events across a wide range of topics, including entertainment, sports, business, politics, and even natural disasters. These findings support the notion that changes in public sentiment toward any major event—cases of scientific misconduct included—might be as much a function of the attention received as it is a function of the theme or merits of any specific case. As the saying goes, “no news is good news”—and this study demonstrates this quite clearly. Once the STAP story becomes tainted by allegations of misconduct, increases in public attention—driven mostly by the public relations (PR) efforts of the respective actors—consistently corresponded with increases in overall negativity. The only event that broke this trend was one of the only events not staged for publicity—the apparent suicide of a key stakeholder. Here, we observed a clear and significant positive shift in overall sentiment; however, this was also accompanied by a notable subsequent decrease in volume. Overall, these results strongly suggest that, in cases of research misconduct, public opinion—and by extension, public policy—is likely to be more influenced by negative-leaning news and reporting. Academic researchers, policy makers, and those with associated interests are advised to carefully consider the implications.

## Appendix

Multimedia Appendix 1 A summary timeline.

## Abbreviations

ANOVA: analysis of variance
ATP: adenosine triphosphate
BBC: British Broadcasting Corporation
CNN: Cable News Network
HSD: honest significant difference
PR: public relations
SNS: social network services
STAP: stimulus-triggered acquisition of pluripotency
SVM: support vector machine
TF-IDF: term frequency-inverse document frequency
