# Supplementary figures and images for: Public Response to Scientific Misconduct: Assessing Changes in Public Sentiment Toward the Stimulus-Triggered Acquisition of Pluripotency (STAP) Cell Case via Twitter
Source: JMIR Public Health Surveill. 2017 Apr 20;3(2):e21. doi: 10.2196/publichealth.5980 (PMC5418527; doi:10.2196/publichealth.5980)

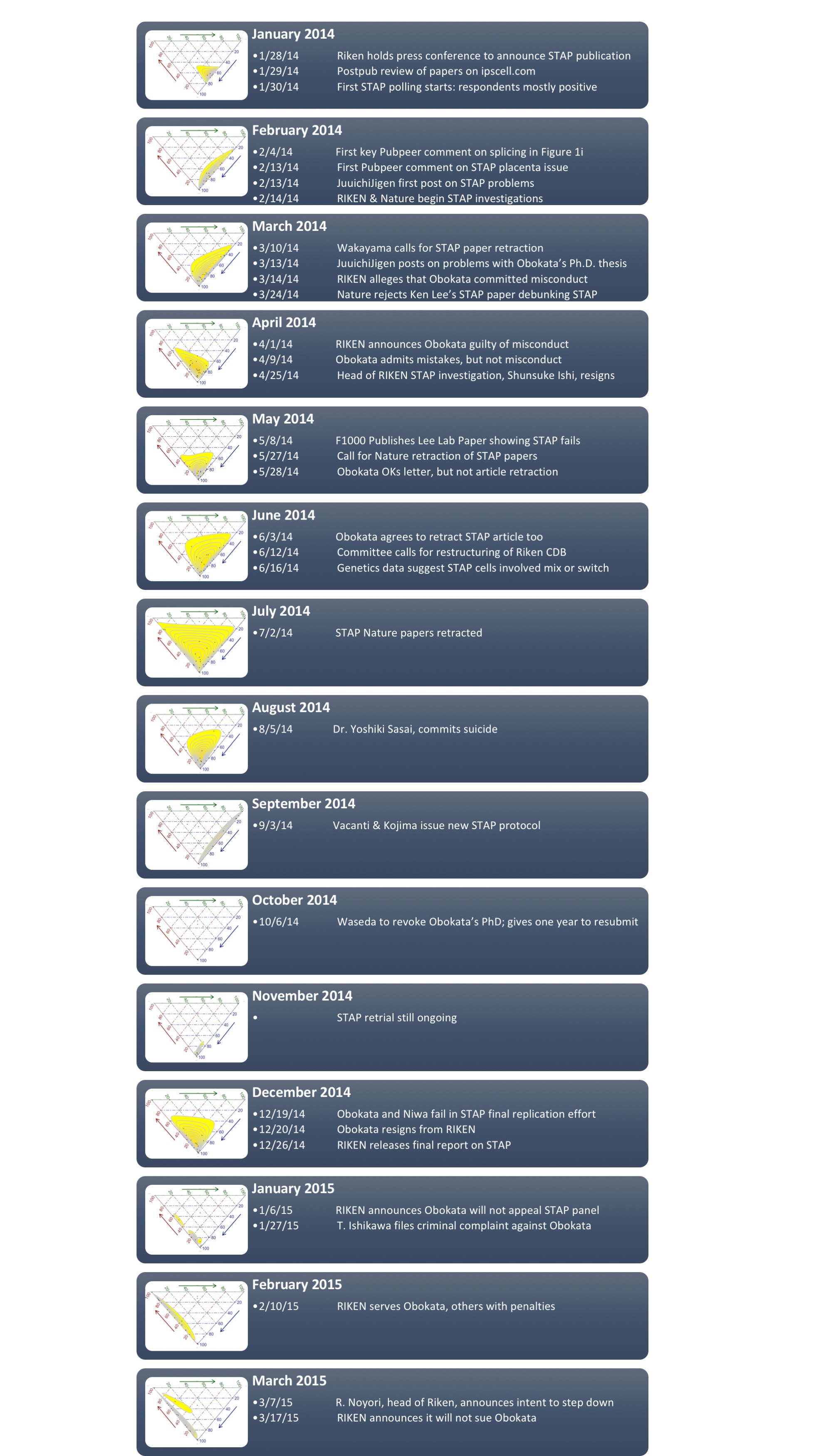

Supplement: Multimedia Appendix 1 [file publichealth_v3i2e21_app1.jpg]
